# Short-Term Outcomes of Laparoscopic Sleeve Gastrectomy for Weight Loss and Gastroesophageal Reflux Disease

**DOI:** 10.7759/cureus.76943

**Published:** 2025-01-05

**Authors:** Mian S Yousaf, Noman Khan, Ghulam Fareed, Arbab M Kashif Khan, Saad Aziz, Masood M Karim

**Affiliations:** 1 Gastroenterology, Prime Teaching Hospital, Peshawar, PAK; 2 Department of Gastroenterology, Prime Teaching Hospital, Peshawar Medical College, Peshawar, PAK; 3 Department of Gastroenterology, Kulsum International Hospital, Islamabad, PAK; 4 Gastroenterology, Peshawar Medical College, Peshawar, PAK; 5 Consultant Gastroenterologist, Prime Teaching Hospital, Peshawar, PAK; 6 Gastroenterology and Hepatology, Aga Khan Health Service, Pakistan, Karachi, PAK

**Keywords:** bariatric surgery, comorbidities, gastroesophageal reflux disease, laparoscopic sleeve gastrectomy, obesity, weight loss

## Abstract

Background and objective

Laparoscopic sleeve gastrectomy (LSG), a novel bariatric technique, reduces stomach capacity to promote weight loss. It is increasingly preferred due to its lower risk and improved outcomes, particularly for obesity-associated hypertension and diabetes. LSG has also demonstrated effective weight loss. This study aimed to evaluate the short-term outcomes of LSG for treating obesity and gastroesophageal reflux disease (GERD).

Methodology

A prospective cohort study was conducted at Lady Reading Hospital-Medical Teaching Institute (LRH-MTI), Peshawar, from March 2023 to February 2024. A total of 280 patients (aged 18-60 years) were selected using a consecutive sampling technique. Weight loss was assessed using the percentage of total body weight loss (%TBWL) and excess weight loss (%EWL). GERD symptoms were evaluated using the GerdQ score. Statistical analyses, including paired t-tests and Chi-squared tests, were performed using SPSS version 26 (IBM Inc., Armonk, New York), with significance set at p<0.05.

Results

LSG achieved a 100% success rate with no major complications, an average surgery duration of 45 minutes, and a mean hospital stay of 2.5 days. At the 12-month follow-up, significant reductions in hypertension (p=0.032) and diabetes (p<0.012) were observed. Substantial weight loss (p=0.001) and a decrease in comorbidities were noted, while GERD symptom improvement was not statistically significant (p=0.348).

Conclusion

LSG is a promising intervention for weight loss and obesity-related comorbidities. It significantly improves hypertension and diabetes, although its impact on GERD requires further research. Longer follow-ups and diverse populations are recommended for future studies.

## Introduction

In the field of bariatric surgery, laparoscopic sleeve gastrectomy (LSG) has generated a lot of interest due to its effectiveness in managing obesity and its relatively less complicated surgical technique [1]. As a variation of the standard sleeve gastrectomy (SG), LSG involves the creation of a tubular gastric channel and a reduction of stomach capacity by 70-80% [2]. This leads to the consumption of a very low number of calories; the patient feels full very early or even all-time round, which is essential for the management of obesity. Obesity has been widely recognized as a world health problem as incidence rates are growing and resulting in the complexity of comorbid diseases, including type 2 diabetes, cardiovascular diseases, hypertension, and some forms of cancer [3, 4]. This is where LSG steps in as a surgical option for persons who may not have seen the intended changes for the better via diet and exercise.

Although the main focus on LSG is obesity, its impact on other health statuses, especially gastroesophageal reflux disease (GERD), has drawn more attention [5, 6]. GERD, a condition in which stomach content moves back into the esophagus, leading to heartburn or regurgitation, is common with obesity. The association between obesity and GERD has been researched extensively, wherein having extra weight leads to increased intra-abdominal pressure, thus leading to worsening reflux symptoms [7, 8].

Thus, while LSG appears to have positive impacts on GERD, the relationship between the two remains more fragmented and thus necessitates further research. Several investigations point to the positive trend of GERD symptoms after LSG, likely due to a reduction in intra-abdominal pressure, while others indicate that LSG leads to worsening or even development of GERD [9, 10]. These differences in outcome could be attributed to the anatomic changes created by LSG about the regulation of the fundus of the stomach and the lower esophageal sphincter [11]. Hence, knowledge of the temporary ramifications of LSG on both weight loss and GERD is crucial for determining which patients are most likely to benefit from the procedure, which approach to surgery is best, and how patients should be treated in the immediate aftermath of the surgery.

In the short term, LSG has effectively achieved weight loss in the first six to twelve months after surgery, with many patients losing between 30-50% of their excessive body weight [12, 13]. Such rapid weight loss can have a positive effect on obesity comorbidities such as insulin resistance, hypertension, and dyslipidemia [14]. Yet, the effect on GERD is still uncertain, and while some studies show improvements in GERD symptoms in patients who initially had the disease, other investigations describe the development of GERD symptoms in those who did not have it before.

The purpose of this study was to examine the short-term implications of LSG with specific attention paid to the effectiveness of the procedure to reduce weight and the impact it has on GERD. This study aims to identify the advantages and possible dangers of LSG so that physicians can make the best decisions on behalf of their patients. Furthermore, these findings can help identify preoperative characteristics that affect GERD symptoms after LSG and direct the design of novel surgical techniques and postoperative management plans to enhance the patient’s quality of life.

Objective

The objective of this study was to assess the short-term results of laparoscopic sleeve gastrectomy for the treatment of obesity and GERD.

## Materials and methods

Study design and study setting

This research was planned as a prospective cohort study that was carried out between March 2023 and February 2024 at Lady Reading Hospital-Medical Teaching Institute (LRH-MTI), Peshawar. The sampling technique adopted was probability sampling; random sampling was applied to recruit participants.

Sample population and sampling criteria

Patients aged 18 to 60 who underwent laparoscopic sleeve gastrectomy (LSG) for the treatment of severe obesity were included in this study. To qualify, participants were required to have a preoperative body mass index (BMI) of at least 35 kg/m² or, if their BMI was below 35 kg/m², at least one obesity-related comorbid condition such as type 2 diabetes, hypertension, or obstructive sleep apnea.

Eligible participants were required to have comprehensive medical records, including both preoperative and postoperative data, a follow-up period lasting at least 12 months after surgery, and documented evaluations of gastroesophageal reflux disease (GERD) symptoms or confirmation of GERD.

Exclusion criteria included individuals who underwent LSG in combination with other bariatric procedures (e.g., gastric bypass), those with incomplete medical records or missing follow-up data, patients with a history of prior gastrointestinal surgeries that could influence the outcomes of LSG (e.g., fundoplication), and those diagnosed with severe gastrointestinal conditions (e.g., active peptic ulcer disease or Barrett's esophagus).

Sample size

There were 384 individuals included in the research's participant sample calculated using the World Health Organization (WHO) formula [15]. However, only 280 participants were included in the study because the rest 104 lost follow-up.

Sample size calculation

The calculation is performed using the WHO formula *n*= *Z ^2^* x *p* x (1 - p) /*d *^2^, where *n* is the required sample size, *Z* is the Z-score for the 95% confidence level, *p* is the point estimate of the prevalence of the outcome (estimated at 50% based on preliminary data), and *d* is the margin of error of 0.05.

Data collection

Information gathering also included chart abstraction of preoperative, perioperative, and postoperative data through a library search of the hospital's electronic medical record system. Collectively, study data at admission encompassed age, gender, BMI, hypertension, diabetes, prior symptoms of GERD, and history of GERD assessed by the BAROS questionnaire. Perioperative data was a report of the successful technique of the LSG procedure and any complications from it. The follow-up data encompassed the weight loss results in terms of percentages of total body weight as well as BMI categories, which were also evaluated postoperatively, as well as potential postoperative complications with preference for the worsening of GERD symptoms.

After one year, an investigator contacted the patients to participate in a telephone conversation along with clinical assessments in addition to the GerdQ score (e.g., endoscopic evaluation and pH testing). Participants were asked about diabetes, hypertension, weight loss, GERD, and the usage of medications to manage blood pressure. The intake and termination of drugs for hypertension and diabetes following surgery were used to quantify the existence and recovery of these medical conditions. A blood sugar level of more than 5.6 mmol/L while fasting and a glycosylated hemoglobin level of 6.5% without the use of insulin or oral hypoglycemic drugs were considered indicators of diabetes recovery. The improvement was described as a reduction in the quantity or dose of oral hypoglycemic drugs or insulin. Recovering from hypertension was defined as achieving a systolic blood pressure below 130 mmHg and a diastolic blood pressure below 85 mmHg without the need for antihypertensive medication. 

GERD symptoms were scored using the recognized scoring systems, including the GERD Questionnaire (GerdQ) score. These three scores were used to categorize the patients to either having worsening, no change, or improvement in their GERD symptoms. The percentage of total body weight loss (%TBWL) and percentage of excess weight loss (%EWL) were used to measure weight reduction after six months and 12 months following the surgery. This information was gathered through regular monitoring and follow-up clinical appointments.

Variables

Continuous variables included age, weight, BMI, percentage of total body weight loss (%TBWL), and percentage of excess weight loss (%EWL). On the other hand, categorical variables were expressed as frequencies and proportions. This included gender and the presence of comorbidities, such as hypertension, diabetes, and gastroesophageal reflux disease (GERD).

Data analysis

Records were kept in Microsoft Excel (Microsoft, Redmond, Washington), and statistical analysis was done using SPSS version 26. Descriptive and inferential statistics were used, and their value was 0. Normally distributed quantitative data of age, sex, BMI, and change in weight were described using means and standard deviations, while non-continuous variables, including the presence of diabetes, hypertension, and GERD symptoms, were described using frequencies and proportions. Descriptive analysis of continuous data, Normality assumes of continuous data were checked using the Shapiro-Wilk test. To analyze differences in weight loss and GERD scores before and after the operation, the variables that had a normal distribution, the paired t-test was used; for data that wasn't normally distributed, the Wilcoxon signed-rank test was used. For analyzing all variables, a p-value of less than 0.05 was taken as the level of significance.

Ethical statement

Ethical approval for this study was provided by the Institutional Review Board (IRB) of Lady Reading Hospital-Medical Teaching Institute (LRH-MTI), Peshawar (Approval No. 77/LRH/MTI, dated Feb 23, 2023). All the participants provided informed consent, and privacy was observed during the entire study process. To protect the identities of the patients, all data collected were made anonymous, and patients were informed of their right to participate without coercion and that they could even cease participating without regret in their medical treatment.

## Results

We conducted the present study from March 2023 to February 2024 and found that 280 patients underwent laparoscopic sleeve gastrectomy (LSG). All the surgeries were performed by the same surgeon. The mean age of the patients at the time of surgery was 45.4 ± 8.5 years. The average weight was 102.3 kg, and the mean body mass index (BMI) was 35.7 ± 4.8. The preoperative assessment revealed that 87 patients (31.1%) complained of gastroesophageal reflux disease (GERD). Every patient had a successful LSG operation with no significant intraoperative problems. The average duration of the hospital stay was 2.5 days, and the average surgery time was 45 minutes. All patients were discharged with postoperative instructions, and none required conversion to open surgery. Follow-up data were gathered, and regular visits were used to monitor each patient's progress. The fundamental preoperative clinical and demographic information of the 280 participants is shown in Table 1.

**Table 1:** Preoperative baseline characteristics of the study population Age is expressed as mean ± standard deviation. Gender distribution, preoperative presence of gastroesophageal reflux disease (GERD), and comorbidities such as hypertension and diabetes are shown as frequencies.

Characteristic	n = 280
Age (years) (mean ± SD)	45.4 ± 8.5
Gender	
Male	121 (43.2%)
Female	159 (56.8%)
Body mass index (BMI) (mean ± SD)	35.7 ± 4.8
Preoperative GERD	87 (31.1%)
Comorbidities	
Hypertension	89 (31.8%)
Diabetes	72 (25.7%)
None	119 (42.5%)

Patients experienced significant reductions in body weight over time, as indicated by the weight loss outcomes (p=0.001). Table 2 presents the weight loss outcomes at six and 12 months, including the percentages of total body weight loss (%TBWL) and excess weight loss (%EWL), with p-values demonstrating the statistical significance of these changes. Regarding GERD, most patients reported improvement at each follow-up interval, though the association was not statistically significant (p=0.348).

**Table 2:** Weight loss outcomes postoperative

Time point	% total body weight loss (TBWL) (mean ± SD)	% excess weight loss (EWL) (mean ± SD)	p-value
6 months	15.4 ± 4.2	31.2 ± 8.5	0.001
12 months	22.5 ± 5.7	46.9 ± 9.6	

The analysis of GERD symptoms before and after LSG revealed notable trends in improvement over time. At the six-month follow-up, 42 patients (15.0%) reported worsening symptoms, 79 patients (28.2%) experienced no change, and 159 patients (56.8%) noted improvement. By the 12-month follow-up, the number of patients with worsened symptoms decreased to 28 (10.0%), those with unchanged symptoms reduced to 71 (25.4%), and the number of patients reporting improved symptoms increased significantly to 181 (64.6%). However, the association between GERD symptom changes and time was not statistically significant (p=0.334), as shown in Table 3.

**Table 3:** GERD symptoms before and after LSG (n = 280) GERD - gastroesophageal reflux disease; LSG - laparoscopic sleeve gastrectomy

GERD symptom status	6 Months (n, %)	12 Months (n, %)	p-value
Worsened	42 (15.0%)	28 (10.0%)	0.334
Unchanged	79 (28.2%)	71 (25.4%)	
Improved	159 (56.8%)	181 (64.6%)	

**Figure 1:**
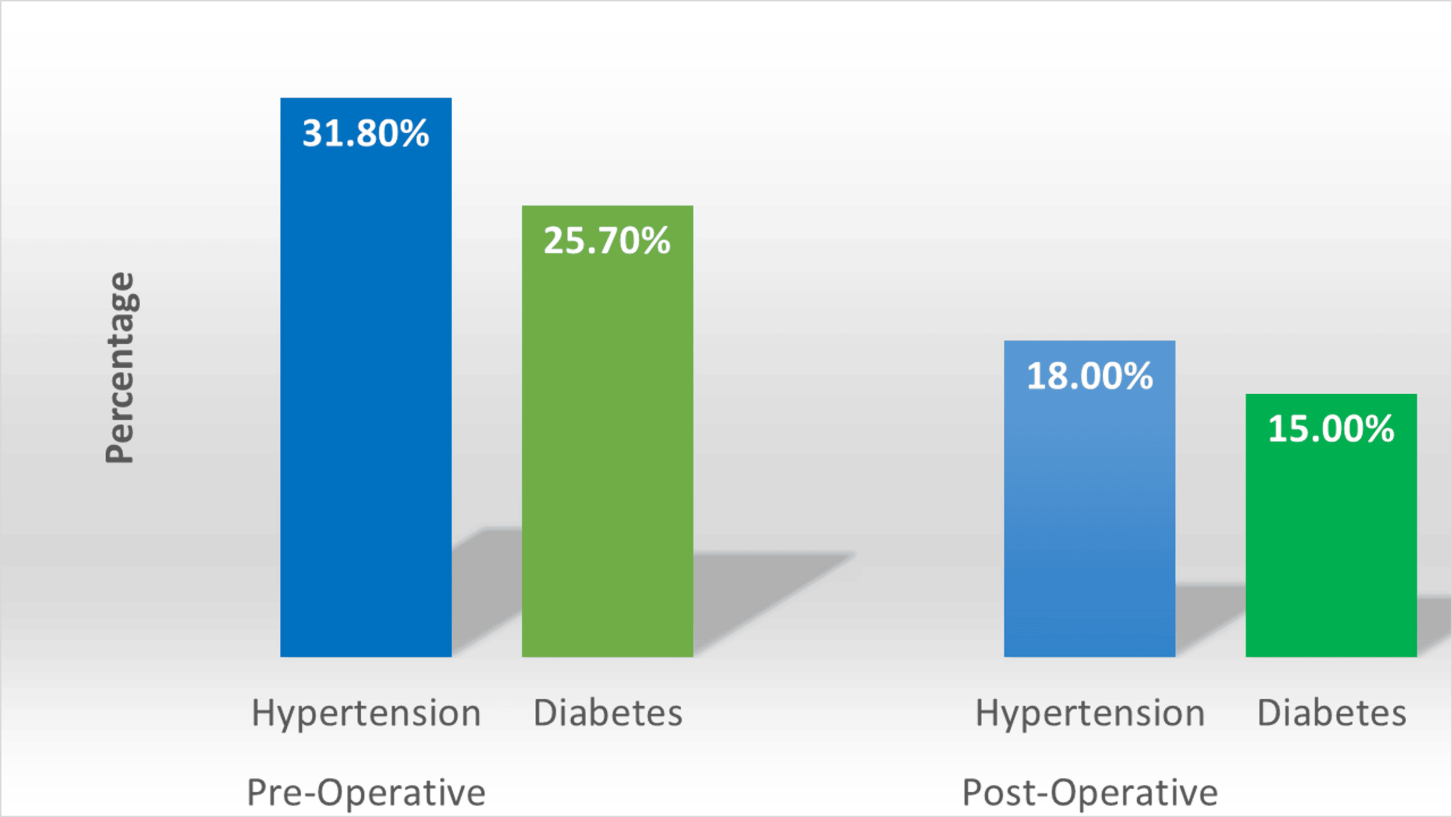
A graph illustrating a notable reduction in the prevalence of hypertension and diabetes at the 12-month follow-up

Figure 1 highlights the significant reduction in the prevalence of hypertension and diabetes at the 12-month follow-up, demonstrating the positive impact of laparoscopic sleeve gastrectomy (LSG) on these comorbid conditions. Before the procedure, the prevalence rates were 31.8% for hypertension (89 participants) and 25.7% for diabetes (72 participants), which decreased to 18.0% (50 participants) and 15.0% (42 participants), respectively, after surgery.

## Discussion

The present research evaluated the experience of laparoscopic sleeve gastrectomy done within one year at our center. Based upon a patient group of 280 patients, all operations performed by one surgeon, our findings provide a clear insight into the efficacy and safety profile of LSG. The mean age of all patients was 45.4 ± 8.5 years and a preoperative average weight of 102.3 kg. In particular, 50% of the patients had gastroesophageal reflux disease (GERD) prior to the surgery. The patients' satisfaction and safety results in the perioperative period were satisfactory; patients did not report significant intraoperative complications, and the rate of successful procedures was 100%. The follow-up data reveal a more substantial lessening of comorbidities and weight loss but only a moderate resolution of GERD symptoms.

Our study reveals surgery duration and hospital stay to be comparable to the results reported by Lopez-Nava et al. (2022), where the average surgery was found to be about 40-50 minutes, and an average hospital stay was estimated to be about two to three days [16]. Regarding the coexisting conditions, our findings are in line with Fiorillo et al. [17] and Correia et al.[18], where a decrease in hypertension and diabetes rates after LSG was found. The reduction rates of hypertensive (25. 7%) and diabetic (16. 1%) subjects in the present study were nearly similar to the study of Baştürk et al. (2020), where they recorded a reduction rate of 24% and 18% for hypertensive and diabetic patients respectively [19]. Similar to our findings, Jain et al. (2022) and Wityk et al. (2020) also observed improvements in overall health status following LSG [13, 20].

While LSG has demonstrated substantial efficacy in weight loss and reduction of comorbidities, long-term nutritional deficiencies present a significant challenge. As highlighted by Mulita et al. [21], deficiencies in hemoglobin, ferritin, and vitamin B12 worsened significantly over six years post-surgery, emphasizing the need for vigilant postoperative monitoring and individualized supplementation strategies to mitigate these risks and sustain long-term health benefits.

A study by Chen et al. (2020) also highlighted substantial weight loss outcomes in LSG patients, which aligns with our findings [22]. Our results are also in accordance with Hu et al. (2021), who reported similar efficacy in weight management with LSG [23]. We found no statistical significance in GERD symptoms post-LSG, which is in contrast with the results from a study by Elzouki et al. (2021), who showed a more pronounced relief in GERD symptoms post-LSG [24]. This difference may be attributed to differences in patient populations or variations in follow-up intervals.

Limitations and strengths

Our study, while providing valuable insights, has several limitations. Firstly, the lack of a control group limits our ability to compare LSG outcomes directly against alternative weight-loss interventions. Furthermore, it's possible that short-term effects or challenges with a delayed onset were not adequately captured by the very limited 12-month monitoring period. The absence of a standardized GERD assessment tool could have affected the consistency of symptom reporting. Finally, the single-surgeon setting, although ensuring consistency, may not reflect the variability in outcomes observed with different practitioners.

Despite these drawbacks, our research highlights how effective LSG is at helping patients lose a large amount of weight and manage associated conditions, including diabetes and hypertension. Patients with obesity may find LSG to be a safe and useful choice for managing their weight, as evidenced by the high success rate of treatments and the absence of significant intraoperative problems. The slight improvement in GERD symptoms emphasizes the need for more studies to enhance the prognosis for those who already have GERD.

## Conclusions

Our study supports the effectiveness of laparoscopic sleeve gastrectomy as a weight-loss and obesity-related comorbidity therapy approach, demonstrating significant reductions in weight, hypertension, and diabetes prevalence while highlighting the need for further investigation into its variable impact on GERD symptoms to optimize patient outcomes. While our study highlights the effectiveness of laparoscopic sleeve gastrectomy in achieving significant weight loss and managing obesity-related comorbidities such as diabetes and hypertension, its limitations include the absence of a control group, a short 12-month follow-up period, lack of standardized GERD assessment, and single-surgeon setting, emphasizing the need for further research to address these gaps.

## Figures and Tables

**Figure 2:**
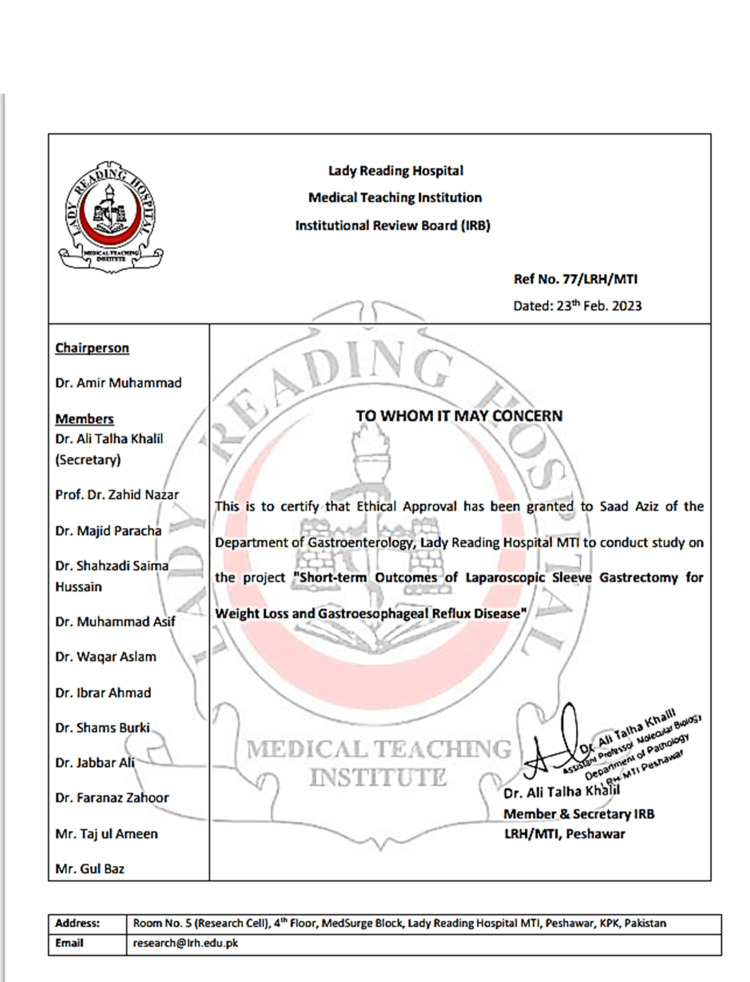
Snapshot of the IRB approval letter
